# Why are the Relatively Deprived Reluctant to Improve Themselves? The Crucial Role of Perceived Upward Economic Mobility

**DOI:** 10.5334/irsp.918

**Published:** 2025-07-09

**Authors:** Zhenzhen Liu, Rongzi Ma, Xiaomin Sun

**Affiliations:** 1 Key Laboratory of Adolescent Cyberpsychology and Behavior (CCNU), Ministry of Education, Wuhan, China; 2 Key Laboratory of Human Development and Mental Health of Hubei Province, School of Psychology, Central China Normal University, Wuhan, China; 3 Beijing Key Laboratory of Applied Experimental Psychology, National Demonstration Center for Experimental Psychology Education (Beijing Normal University), Faculty of Psychology, Beijing Normal University, Beijing, China

**Keywords:** relative deprivation, perceived upward economic mobility, self-improvement

## Abstract

A large number of studies found that relative deprivation leads to destructive behaviors. However, the effects of relative deprivation on behaviors typically deemed constructive, such as self-improvement, remain uncertain. In four studies, the current research provides robust evidence for the negative effect of relative deprivation (X) on self-improvement (Y) and the mediating role of perceived upward economic mobility (M). Specifically, Study 1 (*n* = 220) preliminarily provided correlational evidence for the above mediation model with well-established measurements. Study 2a (*n* = 260) and Study 2b (*n* = 130) applied double randomization designs to manipulate relative deprivation and perceived upward economic mobility separately and showed that direct causal links of each path (X → M, X → Y, and M → Y) existed. Study 3 (*n* = 780) applied blockage manipulation and showed that intervening in perceived upward economic mobility is a potential cure for relative deprivation. The theoretical and practical implications of the results in the current study as well as future research directions are discussed.

Rising income inequality is a global trend in the 21st century (Saez & Zucman 2016). According to the Global Wealth Report 2021, 1% of the global population has 45% of the global wealth. Since people tend to compare themselves with those who are better off when making social comparisons (Gerber, Wheeler & Suls 2018), the widening income gap has made a large portion of the population believe that they are relatively deprived (Liu, Sun & Bao 2024), i.e., the judgment that one is in a disadvantageous situation compared to similar others, which is also accompanied by feelings of anger and resentment (Smith et al. 2012).

What do people do when they feel that they are relatively deprived? Numerous studies have focused on the effect of relative deprivation on destructive behaviors. For example, studies have shown that relative deprivation increases deviant behaviors and escape behaviors: people who experience high level of relative deprivation are likely to commit crimes such as theft and violence (Stiles, Liu & Kaplan 2000), and tend to escape from work (Geurts, Buunk & Schaufeli 1994). Additionally, correlational studies (e.g., Mishra & Novakowski 2016), experimental studies (e.g., Callan et al. 2008), and meta-analysis studies (e.g., Callan, Shead & Olson 2015) have all shown that relative deprivation positively predicts gambling behavior.

In contrast to the abundant research that examines the effect of relative deprivation on destructive behaviors, research on the relationship between relative deprivation and constructive behaviors is scant. However, in the context of high-income inequality, understanding whether and how relative deprivation could affect constructive behaviors is of great practical importance. Participating in self-improvement activities is a typical constructive behavior (Fiske & Berdahl 2007; Guinote 2010), which we defined as behaviors conducted to improve one’s knowledge or skills for the purpose of achieving economic success. The rising income inequality has intensified competition (Cheng, Hao & Wang 2021), causing people to easily fall far behind if they do not continuously improve themselves. Self-improvement helps individuals improve their situation and mitigates the unfavorable effects of high-income inequality on individuals in the long run.

However, some indirect evidence suggests that relative deprivation seems to prevent individuals from engaging in self-improvement activities. For example, scholars who experience relative deprivation are less involved in academic activities (Wosinski 1988). Students who experience relative deprivation report less school enrollment and worse academic achievement (Destin & Oyserman 2009; Esposito & Villasenor 2019; Nieuwenhuis & Chiang 2021). The current study aimed to clarify how self-improvement is affected by relative deprivation. We proposed that relative deprivation decreases self-improvement by decreasing people’s beliefs that their current unfavorable circumstances are changeable.

## The Mechanism Behind How Relative Deprivation Affects Self-Improvement

### Relative deprivation impedes perceived upward economic mobility

Individuals who perceive themselves as highly deprived experience greater subjective disadvantage compared to those who feel minimally deprived. Such perceptions often lead individuals to view their circumstances as unfair (Smith et al. 2012), reinforcing beliefs about inhabiting an unjust world and attributing their disadvantaged position to an inequitable social system (Zheng et al. 2023). Consequently, individuals confronting adversity tend to attribute their unfavorable conditions to factors beyond their control (Crocker et al. 1999; Davidai 2018; Davidai 2023), undermining their belief in the effectiveness of their personal efforts (Davidai 2018). This belief diminishes their expectation of being able to change their circumstances and achieve upward mobility (Li et al. 2019). Therefore, individuals experiencing substantial subjective disadvantage may understandably perceive limited opportunities for improving their situations. Accordingly, we propose that relative deprivation negatively impacts individuals’ perceptions of their potential for upward economic mobility.

The relationship between relative deprivation and perceived upward economic mobility is substantiated by a robust body of empirical evidence. For instance, numerous studies have identified a negative correlation between relative deprivation and future expectations, a construct intricately linked to perceived upward mobility (e.g., Chen et al. 2018; Feldman, Leana & Bolino 2002; Gray & Dagg 2019). For example, Feldman et al. (2002) found that relative deprivation negatively influenced individuals’ attitudes toward their future careers and Nieuwenhuis & Chiang (2021) noted its adverse effects on students’ educational aspirations.

Furthermore, economic inequality, which frequently precedes relative deprivation (Liu, Sun & Bao 2024; Osborne, García-Sánchez & Sibley 2019), has been consistently associated with diminished expectations of upward economic mobility (Browman, Destin & Miele 2022; Davidai 2018; Melita, Rodriguez-Bailon & Willis 2023). This converging evidence underscores the detrimental impact of relative deprivation on individuals’ perceptions of their potential for economic advancement.

### Lower perceived upward economic mobility hinders individuals’ propensity to engage in self-improvement activities

Individuals with low perceived upward economic mobility are likely to believe that even substantial efforts will not enable them to reach the economic status of those who are better off (Yoon & Kim 2016). This diminished expectation often translates into a loss of hope, where individuals doubt their ability to achieve positive and successful economic outcomes through their own efforts.

Expectancy theory (Vroom 1964) suggests that when personal efforts are perceived as unlikely to achieve desired goals, motivation decreases, leading to inaction or even self-handicapping behaviors (e.g., Shi & Huang 2004). This theory is borne out in empirical studies, such as one involving middle school students (Browman et al. 2017). This study demonstrated that students who believed hard work would not lead to success (low upward mobility belief condition) were less likely to persevere in the face of academic challenges and subsequently received lower grades compared to their peers in higher mobility belief or control conditions.

The primary aim of the current study is to empirically examine how relative deprivation influences self-improvement behaviors and to elucidate the mediating role of perceived upward economic mobility. Specifically, we proposed the following hypotheses:

**Hypothesis 1**: Relative deprivation negatively predicts participation in self-improvement activities.

**Hypothesis 2**: Perceived upward economic mobility mediates the relationship between relative deprivation and self-improvement, such that relative deprivation diminishes self-improvement efforts through its negative impact on perceived upward economic mobility.

## The Present Research

The current research comprised four studies designed to test the proposed hypotheses. In Study 1, the relationships among relative deprivation, self-improvement behaviors, and perceived upward economic mobility were preliminarily explored based on data collected using well-established questionnaires. Studies 2a and 2b employed double randomization designs to gather further causal evidence supporting the mediation effect. Study 3 utilized a blockage design to manipulate relative deprivation and perceived upward economic mobility, examining whether adjustments in perceived upward economic mobility could mitigate the negative effects of relative deprivation. The current research received ethical approval from the Academic Ethics Committee of the Faculty of Psychology at the corresponding author’s institution before the commencement of the study.

### Study 1

#### Methods

##### Participants

We conducted an *a priori* power analysis using the ‘shiny’ application developed by Schoemann et al. (2017) to determine the appropriate sample size required for our mediation analysis. The result indicated that 160 participants would provide a power of at least 80% to detect the mediating effect, assuming the correlations between relative deprivation, perceived upward economic mobility, and self-improvement intentions are all small-to-medium (assuming the correlation matrix is: *r*_RD-PEM_ = –0.30; *r*_RD-SI_ = –0.30; *r*_PEM-SI_ = 0.30; and SDs = 1). Ultimately, 220 Chinese participants (35.9% were male and 64.1% were female, *M*_age_ = 30.96 years, *SD* = 5.78) who were employed full-time were recruited via an online crowdsourcing platform (www.Credamo.com). All participants provided consent online before completing the questionnaire.

#### Materials

##### Relative deprivation

We used a 7-item scale (Cronbach’s α = 0.92) adapted from the Personal Relative Deprivation Scale (Callan, Shead & Olson 2011) and the Relative Deprivation Questionnaire (Ma 2012). A sample item is ‘I feel dissatisfied with what I have compared to what other people like me have.’ The participants rated each item using a 7-point Likert-type scale from 1 (strongly disagree) to 7 (strongly agree).

##### Perceived upward economic mobility

We adapted the Perceived Social Mobility Scale (Day & Fiske 2017) by substituting ‘economic class/economic’ for ‘social class/social’ to measure perceived upward economic mobility. The scale comprised 8 items (Cronbach’s α = 0.92). A sample item is ‘There are a lot of opportunities for people to move up the economic ladder (1 = strongly disagree, 7 = strongly agree)’. Each item was rated on a 7-point Likert-type scale.

##### Self-improvement

Self-improvement was assessed using an adapted version of the Participation in Employee Development Activities Scale (Hurtz & Williams 2009). The original scale includes four dimensions, employee assessment, on-the-job experiences, courses and programs, and professional relationships. Based on the existing literature (Birdi, Allan & Warr 1997; Zoogah 2010), we added a fifth dimension, career planning. Consequently, the finalized scale encompasses five dimensions, each containing 4, 4, 5, 4, and 4 items respectively. A sample item is ‘In the next 12 months, I want to seek feedback from my supervisor about my job-related behaviors’ Responses were collected using a 7-point Likert-type scale, ranging from 1 (not likely at all) to 7 (most likely). The internal consistency for each dimension was satisfactory, with Cronbach’s α values ranging from .73 to .83.

##### Demographic variables

Control variables included gender (male = 0, female = 1), age, education level (senior high school = 1, junior college = 2, bachelor’s degree = 3, and master’s degree or above = 4), and subjective socioeconomic status (SSES) were collected. SSES was assessed by the MacArthur Scale of Subjective Social Status. This scale features a 10-rung ladder visual representation (Adler et al. 2000).

All scales were translated into Chinese following a rigorous back-translation procedure when a Chinese version was not available. This translation process was consistently applied in subsequent studies. Information on the construct validity of each scale in Study 1 is available in the supplementary materials.

#### Results and Discussion

##### Common method bias

The results of the common method bias test indicated that the effect was nonsignificant (details provided in supplementary materials).

##### Correlations

The results, as detailed in Table 1, indicated that relative deprivation was negatively related to perceived upward economic mobility (*r* = –.51, *p* < .001) and overall self-improvement (*r* = –.25, *p* < .001). Perceived upward economic mobility showed a positive correlation with self-improvement (*r* = .43, *p* < .001).

**Table 1:** Correlations and Descriptive Statistics (Study 1).


	1	2	3	4	5	6	7	8	9	10	11	12

1. RD	–											

2. PUEM	–.51***	–										

3. SI	–.25***	.43***	–									

4. SI1	–.28***	.38***	.72***	–								

5. SI2	–.20**	.40***	.85***	.54***	–							

6. SI3	–.22**	.21**	.78***	.48***	.54***	–						

7. SI4	–.12	.36***	.82***	.58***	.70***	.46***	–					

8. SI5	–.22**	.42***	.86***	.47***	.66***	.65***	.60***	–				

Covariates												

9. SSES	–.31***	.39***	.16*	.14*	.18**	.09	.09	.15*	–			

10. Gender	–.06	–.02	–.003	–.06	–.04	.10	–.09	.05	–.04	–		

11. Age	–.25***	.21**	.08	.16*	.04	–.05	.17*	.05	.26***	–.05	–	

12. Education	–.12	.03	.08	.10	.04	.09	.04	.06	.03	.02	.05	–

*M*	3.17	4.74	5.72	5.87	5.70	5.93	5.73	5.32	5.66	–	30.96	3.06

*SD*	1.25	1.21	0.70	0.67	0.91	0.77	0.91	1.08	1.05	–	5.78	0.42


*Notes*. RD: relative deprivation, PUEM: perceived upward economic mobility, SI: self-improvement, SI1: employee assessment dimension, SI2: on-the-job experiences dimension, SI3: courses and programs dimension, SI4: professional relationships dimension, SI5: career planning dimension. *M*: mean. *SD*: standard deviation. *p < .05, **p < .01, ***p < .001.

##### Test of the mediating role of perceived upward economic mobility

To evaluate the mediating effect of perceived upward economic mobility, we employed *Mplus* 8 and used Structural Equation Model (SEM). We applied the item parceling method to reduce estimation bias associated with too many indices for the latent variable (Wu & Wen 2011). Specifically, we adopted the random method, which is a common approach for one-factor item parceling (Rogers & Schmitt 2004), to create three parcels for relative deprivation and perceived upward economic mobility. For self-improvement, we adopted the internal consistency approach, which is a commonly used method to deal with multidimensional item sets (Little et al. 2002), to create five parcels using their facets as grouping criteria.

**Figure 1:**
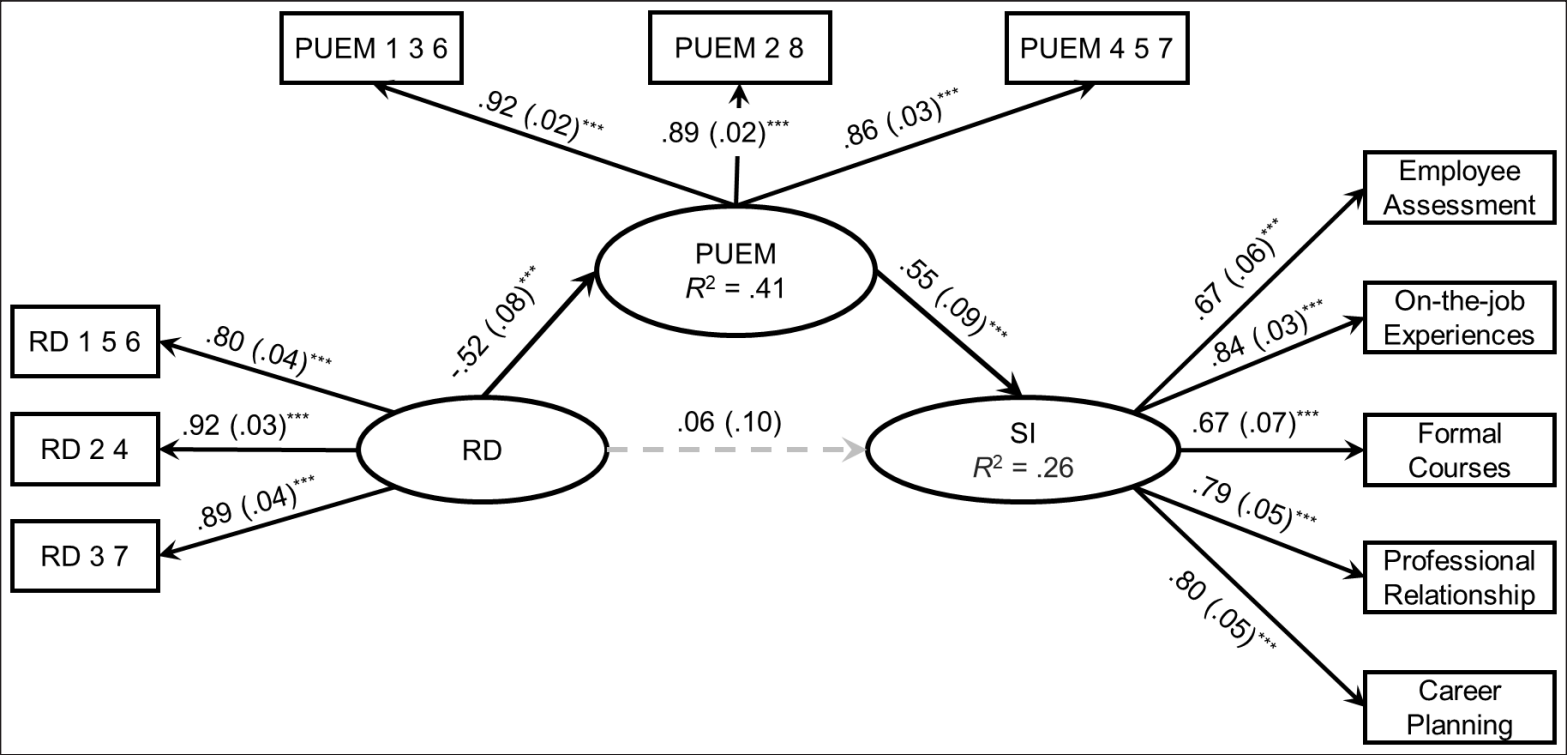
Mediation Model (Study 1). *Notes*. The above path coefficients were all standardized. Black solid lines indicate that the pathways were significant. Values in parentheses are SE. Control variables were SSES, gender, education, and age. RD: relative deprivation, PUEM: perceived upward economic mobility, SI: self-improvement. ***p < .001.

For the specific analysis strategy, following the recommendation of Yzerbyt et al. (2018) related to test mediating effects, we first examined the significance of component paths of ‘X → M’ and ‘M → Y’ employing the joint-significant test, and then examined the magnitude and confident interval of indirect effect using percentile bootstrap method with 5000 resamples. The model fit the data well: χ^2^ (73) = 189.92, *p* < .001; RMSEA = .085 with a 90% CI = [.07, .10]; CFI = .93; TLI = .91; and SRMR = .05. The total effect of relative deprivation on self-improvement was negative and significant (β = –.22, SE = .09, *p* = .01), supporting Hypothesis 1. Regarding the mediating effect of perceived upward economic mobility, as shown in Figure 1, relative deprivation negatively and significantly predicted perceived upward economic mobility (β = –.52, SE = .08, *p* < .001), and perceived upward economic mobility positively and significantly predicted self-improvement (β = .55, SE = .09, *p* < .001). Those results indicated that the indirect effect of perceived upward economic mobility was significant. The mean value of the indirect effect was –.29, with a 95% CI ranging from –.45 to –.16. Thus, Hypothesis 2 was supported.

##### Additional analysis

Given the high correlation observed between relative deprivation and perceived upward economic mobility, we calculated a collinearity diagnostic by calculating the Variance Inflation Factor (VIF). The VIF is determined using the formula VIF = 1/(1 – *R*^2^). For example, when calculating the VIF of perceived economic upward mobility, perceived upward economic mobility was set as the dependent variable, with relative deprivation and control variables as the independent variables. The resultant *R*^2^ was 0.41, leading to a VIF for perceived upward economic mobility of 1.20. This process was repeated for other variables. All calculated VIF values were below the commonly used threshold of 10, indicating that multi-collinearity does not pose a significant concern in our analysis.

While Study 1 demonstrated the negative effect of relative deprivation on self-improvement intentions and the mediating role of perceived upward economic mobility, it must be noted that the relationship identified was correlational. To address this and provide robust causal evidence, Study 2a and Study 2b employed double randomization designs (Pirlott & MacKinnon 2016). In Study 2a, relative deprivation was experimentally manipulated to assess its direct causal effects on both perceived upward economic mobility and self-improvement (i.e., X➔M and X➔Y). Subsequently, Study 2b was designed to manipulate perceived upward economic mobility while controlling for relative deprivation. This allowed us to isolate and examine the specific causal impact of perceived upward economic mobility on self-improvement (i.e., M➔Y, with controlled X). Together, these studies directly tested each pathway (X➔M, X➔Y, and M➔Y), providing a comprehensive evaluation of the causal links among the variables.

### Study 2a

The preregistration details for Study 2a are available at https://aspredicted.org/CP6_ZKD. Building on findings from prior research, relative deprivation can undermine individuals’ self-evaluation (Osborne, Sibley & Sengupta 2015) due to the perception of being in an inferior situation compared to others. In this study, the manipulation of relative deprivation leads participants to experience varying degrees of disadvantage across different experimental conditions, which may subsequently threaten their self-esteem.^1^ Given the potential impact of self-esteem on self-improvement behaviors (Mochon, Norton & Ariely 2012), we included self-esteem as a covariate in our analysis to account for its confounding effects.

#### Methods

##### Participants

A priori power analysis revealed that 128 participants were needed to have adequate power (1 – β = .80) to detect the manipulation effect (using G*power, assuming a medium effect size of *f* = .25 (i.e., partial η^2^ = .06), and α = .05; Faul et al., 2009) using one-way ANOVA. To detect the mediation effect using the ‘shiny’, assuming a small-to-medium correlation (assuming the correlation matrix is: *r*_RD-PEM_ = –.30; *r*_RD-SI_ = –.30; *r*_PEM-SI_ = .30; and SDs = 1) with 80% power, 160 participants were needed. Ultimately, we recruited 260 participants (26.2% male, *M*_age_ = 28.03 years, *SD* = 7.85) via the same online platform that we used in previous studies.

##### Procedure and materials

After providing their online consent form, participants were randomly assigned to either the high relative deprivation (high-RD) condition (*n* = 130) or the low relative deprivation (low-RD) condition (*n* = 130). They completed measures of manipulation check, perceived upward economic mobility, self-improvement, self-esteem, attention checks, and demographic variables. All items of the measures of manipulation check items, perceived upward economic mobility, and self-esteem were rated on a 7-point Likert-type scale (1 = strongly disagree, 7 = strongly agree).

##### Manipulation of relative deprivation

The year-end bonus paradigm was employed to manipulate relative deprivation given that its effectiveness among student samples has been confirmed in previous studies (Sim et al. 2018). Participants in the high-RD condition were instructed to imagine receiving a bonus of 30,000 CNY, which was half the amount—60,000 CNY—received by their co-workers in the same department and position. Conversely, those in the low-RD condition were asked to imagine that everyone in their department and position received an equal bonus of 30,000 CNY. To enhance the manipulation’s effectiveness, participants were then prompted to write down their thoughts and feelings upon learning about their bonus amount.

##### Manipulation check

Four items modified from the Personal Relative Deprivation Scale (Callan, Shead & Olson 2011) were used as the manipulation check items (Cronbach’s α = .96; e.g., ‘Considering the year-end bonuses of my colleagues, I feel dissatisfied with the bonus I received.’)

##### Perceived upward economic mobility

Perceived upward economic mobility in the imaginary organization was measured by a 4-item scale (Cronbach’s α = .96) adapted from the economic mobility scale used in the study by George, Chattopadhyay, and Zhang (2012). A sample item is ‘It is possible for me to get a job with higher payment within this organization.’

##### Self-improvement

Consistent with Study 1, self-improvement in Study 2a was assessed across five dimensions: employee assessment, on-the-job experiences, courses and programs, professional relationships, and career planning (Birdi, Allan & Warr 1997; Hurtz & Williams 2009; Zoogah 2010). Adapting from the approach used by Overall et al. (2010), we employed a 10-item (Cronbach’s α = .96) scale to measure both the participants’ willingness and their potential efforts towards self-improvement, with two items dedicated to each dimension. Example items included, ‘To what extent do you want to improve your work skills in the current company? (1 = not at all, 7 = very much)’ and ‘How much effort are you willing to put into improving your work skills in the current company? (1 = very little effort, 7 = a lot of effort).’ Higher average scores indicated greater engagement in self-improvement activities.

##### Self-esteem

It was measured using four items (Cronbach’s α = .88; e.g., ‘I take a positive attitude toward myself’) adapted from the Rosenberg Self-Esteem scale (Rosenberg 1965).

##### Demographic variables

Demographic information was collected from participants, including gender (male = 1, female = 0), age, SSES (the same as Study 1), and education level (primary school or below = 1, middle school or technical secondary school = 2, high school = 3, junior college = 4, university = 5, master’s degree = 6, and doctoral degree = 7).

##### Results and discussion

Table 2 shows the means, standard deviations, and correlations among the study variables.

**Table 2:** Correlations and Descriptive Statistics (Study 2a).


VARIABLE	1	2	3	4	5	6	7	8	9

1. Conditions	–								

2. Manipulation check	.89***	–							

3. PUEM	–.46***	–.50***	–						

4. Self-improvement	–.25**	–.31***	.75***	–					

5. Self-esteem	–.10	–.12	.22***	.23***	–				

6. SSES	–.02	–.09	.10	.15*	.17**	–			

7. Gender	–.05	–.07	.05	.07	.09	–.08	–		

8. Age	–.03	–.11	.08	.22***	.14*	.22***	.11	–	

9. Education	.04	.08	–.11	–.16*	.00	.16*	–.03	–.16**	–

*M*	–	4.24	4.44	4.80	5.64	5.40	–	28.03	5.08

*SD*	–	2.02	1.53	1.41	0.96	1.30	–	7.85	0.74


*Notes*. PUEM: perceived upward economic mobility; *p < .05, **p < .01, ***p < .001.

##### Manipulation check

An independent sample *t*-test revealed that participants in the relative deprivation condition (*M* = 6.04, *SD* = 0.83) experienced significantly higher levels of relative deprivation compared to those in the control condition (*M* = 2.44, *SD* = 0.98), *t*(258) = 31.90, *p* < .001, Cohen’s d = 3.96 with a 95% CI = [3.71, 4.20]. This finding confirms that relative deprivation was successfully manipulated.

##### The main effect of relative deprivation on self-improvement

The results of the independent-sample *t*-test indicated that participants in the high-RD condition (*M* = 4.45, *SD* = 1.63) reported significantly lower levels of self-improvement compared to those in the low-RD condition (*M* = 5.14, *SD* = 1.03), *t*(258) = –4.10, *p* < .001, Cohen’s *d* = –0.51, with a 95% CI = [–0.75, –0.26]. Furthermore, this effect remained significant after controlling for self-esteem and demographic variables, *F*(1, 253) = 14.20, *p* < .001, partial η^2^ = .05, with a 90% CI = [.02, .10]. These results supported Hypothesis 1.

##### Test of the mediating effect of perceived upward economic mobility

**Figure 2:**
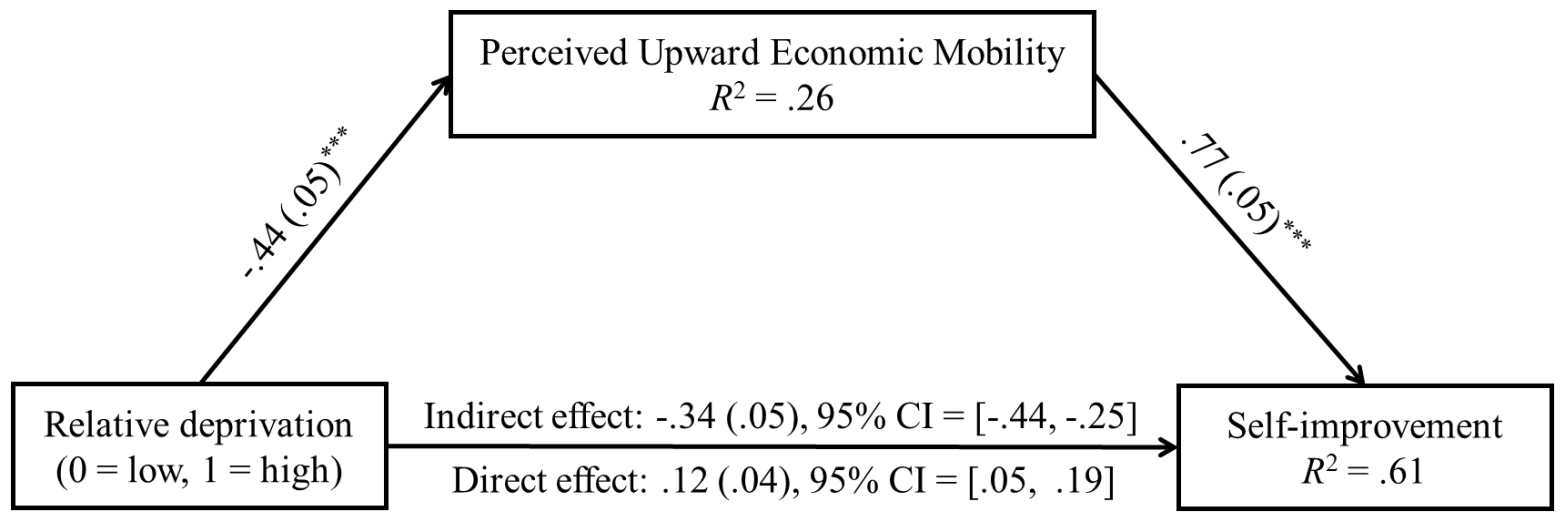
Mediation Model (Study 2a). *Notes*. The above path coefficients were standardized. Control variables were self-esteem, gender, age, education, and SSES. ***p < .001.

A Mediation analysis was performed according to the strategy recommended by Yzerbyt et al. (2018), with self-esteem and demographic variables included as control variables. The results, illustrated in Figure 2, showed that relative deprivation had a significant negative impact on perceived upward economic mobility, β = –.44 with a 95% CI = [–.55, –.33], SE = .05, *t* = –8.04, *p* < .001. Perceived upward economic mobility positively and significantly influenced self-improvement, β = .77 with a 95% CI = [.68, .86], SE = .05, *t* = 16.83, *p* < .001. These findings suggest a substantial mediating effect of perceived upward economic mobility. A percentile bootstrap analysis with 5000 resamples confirmed the significance of the mediating effect, which was quantified at –.34 with a 95% CI = [–.44, –.25]. These results supported Hypothesis 2.

##### Additional analysis

Given the high correlation among relative deprivation, perceived upward economic mobility, and self-improvement, we conducted a collinearity diagnostic following the same methodology as in Study 1. The results showed that all VIF values were below 10, suggesting no significant concern regarding multi-collinearity.

Through the manipulation of relative deprivation, Study 2a provides causal evidence for the negative impact of relative deprivation on both perceived upward economic mobility and self-improvement. Additionally, Study 2b offers further causal support for the effect of perceived upward economic mobility on self-improvement by manipulating perceived upward economic mobility while controlling for relative deprivation.

### Study 2b

The preregistration for Study 2b can be found at https://aspredicted.org/NDB_6F2.

#### Methods

##### Participants

A priori power analysis was conducted using G*Power 3.1 (Faul et al., 2009) to determine the sample size required to assess the effect of manipulation conditions on self-improvement intentions using one-way ANOVA. Assuming a medium effect size of *f* = .25, a power of 80%, and an α = .05, the analysis indicated a required sample size of *N* = 128. Consequently, a total of 130 participants (25.4% male, *M*_age_ = 32.03 years, *SD* = 6.81) were recruited using the same method as in Study 2a.

##### Procedure and materials

After providing online consent, participants first completed measures of relative deprivation. They were then randomly assigned to one of the two experimental conditions: the high perceived upward economic mobility (high-PUEM) condition (*n* = 66) and the low perceived upward economic mobility (low-PUEM) condition (*n* = 64). Following this, participants completed measures for manipulation checks, self-improvement, attention checks, and demographic variables. Finally, participants were debriefed.

##### Manipulation of perceived upward economic mobility

Following the procedure of Shariff et al. (2016), perceived upward economic mobility was manipulated by asking participants to read a report of a survey conducted by Parking University. This report presented differing findings, describing China’s current economic mobility as either high (high-PUEM condition) or low (low-PUEM condition). To reinforce the manipulation, participants were then asked to write at least 30 words reflecting their views on the current state of economic mobility in the country.

##### Manipulation check

Four items from the scale used in Study 1 served as manipulation check items (Cronbach’s α = .94). A sample item is ‘There are a lot of opportunities for people to move up the economic ladder (1 = strongly disagree, 7 = strongly agree)’. All items were rated on a 7-point Likert-type scale.

##### Self-improvement

Self-improvement was measured using two instruments. The first was similar to the one used in Study 2a and consisted of 10 items (Cronbach’s α = .95). Sample items include, ‘To what extent do you want to improve your work skills? (1 = not at all, 7 = very much)’ and ‘How much effort are you willing to put into improving your work skills? (1 = very little effort, 7 = a lot of effort).’ Higher average scores indicated greater self-improvement intentions.

The second measure employed a training program self-enrollment scenario to assess participants’ self-improvement behavior. Participants were informed that a job-related skill training program was open for registration, with previous attendees reporting that the program was well-designed and beneficial for skill enhancement and career development. However, participants were required to pay for the program themselves if they chose to enroll. Two items were adapted from Kurman (2011): one measured the perceived necessity of attending the training, and the other measured willingness to sign up for the training program. These two items were averaged (*r* = .82) to represent the level of self-improvement behavior, with higher scores indicating a higher level of self-improvement behavior.

##### Relative deprivation

The scale used to measure relative deprivation was identical to that in Study 1 (Cronbach’s α = .90).

##### Demographic variables

The demographic variables measured in this study were identical to those in Study 2a.

#### Results and discussion

##### Manipulation check

An independent-sample *t*-test indicated that participants in the high-PUEM condition (*M* = 5.38, *SD* = 1.02) perceived a significantly higher level of upward economic mobility compared to those in the low-PUEM condition (*M* = 2.56, *SD* = 1.14), *t*(128) = 14.83, *p* < .001, Cohen’s *d* = 2.60 with a 95% CI = [2.26, 2.95]. These results suggest that the manipulation of perceived upward economic mobility was successful.

##### The effect of perceived upward economic mobility

After controlling for relative deprivation, one-way ANOVA results revealed a significant main effect of the manipulation conditions on self-improvement. As expected, participants in the high-PUEM condition (*M* = 5.94, *SD* = 0.62) reported higher self-improvement compared to those in the low-PUEM condition (*M* = 5.25, *SD* = 1.31), *t*(128) = 3.86, *p* < .001, Cohen’s *d* = 0.68 with a 95% CI = [0.33, 1.02]. Additionally, they exhibited greater willingness and perceived necessity to participate in the training program (*M_high_* = 5.61, *SD* = 1.33 vs. *M_low_* = 4.63, *SD* = 1.63), *t*(128) = 3.76, *p* < .001, Cohen’s *d* = 0.66 with a 95% CI = [0.31, 1.01]. These findings persisted even when relative deprivation was not controlled for. Specifically, for self-improvement intentions, *F*(1, 127) = 12.60, *p* < .001, partial η^2^ = .09, 90% CI = [.03, .18]; and for participation in the training program, *F*(1, 127) = 11.15, *p* = .001, partial η^2^ = .08, 90% CI = [.02, .17].

In line with the results of Study 1, Studies 2a, and 2b provided causal evidence confirming that relative deprivation decreases individuals’ perceived upward economic mobility, which subsequently harms self-improvement. These findings underscore the crucial role of perceived upward economic mobility in the relationship between relative deprivation and self-improvement, thereby supporting Hypothesis 1 and 2. We hypothesized that if perceived upward economic mobility were maintained at similar levels in both the high-RD and low-RD conditions, the negative impact of relative deprivation on self-improvement would weaken or potentially disappear. Consequently, Study 3 adopted an alternative strategy—Testing-a-Process-hypothesis-by-an-Interaction Strategy (Jacoby & Sassenberg 2010), to investigate whether intervening in perceived upward economic mobility could serve as a remedy for relative deprivation.

### Study 3

The preregistration for Study 3 can be found at https://aspredicted.org/VZY_Y5B. In Study 3, we employed a manipulation-of-mediation-as-a-moderator design (Ge 2023), utilizing a 2 (RD: high vs. low) × 3 (PUEM: high vs. low vs. control) between-subjects experimental design. According to Pirlott & MacKinnon (2016), the high-PUEM and low-PUEM conditions serve as mediator-blocking settings, where the mediator is restricted from varying freely with changes in the independent variable (X). This arrangement eliminates systematic variance in the mediator, leading to a decreased or null effect of X on the dependent variable (Y), depending on whether the mediator partially or fully mediates the relationship between X and Y. Conversely, the PUEM-control condition allows the mediator to vary as expected, resulting in observable mean differences in Y based on X. By considering these two settings simultaneously, our research aims to determine whether the effect of X on Y is contingent upon the blocking of the mediator.

In the current study, in the mediator-blocking conditions (i.e., high- and low-PUEM conditions), the effect of relative deprivation on self-improvement is expected to diminish significantly, potentially to the point of becoming non-significant. Conversely, in the PUEM-control condition, relative deprivation is anticipated to have a significant and negative impact on self-improvement. Additionally, self-esteem will be treated as a control variable, consistent with the approach in Study 2a.

#### Methods

##### Participants

Given that our design can be regarded as a 2 (RD: high vs. low) × 2 (PUEM: high vs. control) and a 2 (RD: high vs. low) × 2 (PUEM: low vs. control) between-subjects design, we determined the sample size based on recommendations from Sommet et al. (2023). They indicated that for a 2 × 2 between-subjects design, if the simple slope of the effect of X on Y is 0.50 in one condition and non-significant (i.e., 0.00) in the other condition, the total required sample size is 502 to test the overall interaction effect with 80% power. This equates to approximately 126 participants per condition. Therefore, we aimed to recruit at least 126 participants in each condition, targeting a total sample size of 756 (i.e., 126 × 6) after exclusions.

Ultimately, we recruited 780 Chinese participants (27.6% male, *M*_age_ = 29.19 years, *SD* = 7.32) with 130 participants in each condition through the same online platform used in previous studies.

##### Procedure and materials

After providing their online consent, participants were randomly assigned to one of the six experimental conditions in a 2 (RD: high vs. low) × 3 (PUEM: high vs. low vs. control) between-subjects design. Firstly, relative deprivation was manipulated, followed by the manipulation of perceived upward economic mobility. Participants then completed measures of self-improvement and self-esteem, with all items rated on a 7-point Likert-type scale. Finally, demographic variables were collected.

##### Manipulation of relative deprivation

Relative deprivation was manipulated in the same way as in Study 2a. The manipulation check items (Cronbach’s α = .97) were also identical to those in Study 2a.

##### Manipulation of perceived upward economic mobility

Perceived upward economic mobility was manipulated within the context of relative deprivation. The paradigm adapted from Day and Fiske (2017) was employed. Participants in the high-PUEM condition were informed that most employees (66%) in this company had improved their relative earnings over the past few years. In contrast, those in the low-PUEM condition were told that the relative income of most employees (66%) has remained largely unchanged. Participants in the control condition received no information regarding economic mobility. The manipulation check items were identical to those used to measure perceived upward economic mobility in Study 2a (Cronbach’s α = .95).

*Self-improvement* (Cronbach’s α = .97), *Self-esteem* (Cronbach’s α = .88), and *Demographic variables* were assessed in the same manner as in Study 2a.

#### Results and discussion

Following Ge (2023) regarding the manipulation-of-mediation-as-a-moderator design, we conducted the following analyses.

##### Manipulation check for relative deprivation

An independent-sample *t*-test suggested that participants in the high-RD condition (*M* = 6.09, *SD* = 0.84) reported significantly higher feelings of relative deprivation compared to those in the low-RD condition (*M* = 2.32, *SD* = 0.97), *t*(778) = 57.94, *p* < .001, Cohen’s *d* = 4.15, with a 95% CI = [4.01, 4.29]. This result confirms that the manipulation of relative deprivation was successful.

##### Manipulation check for perceived upward economic mobility

A one-way ANOVA revealed a significant main effect of the manipulation of perceived upward economic mobility, *F*(2, 777) = 122.29, *p* < .001, partial η^2^ = .24 with a 90% CI = [.20, .28]. Post-hoc comparisons indicated that participants in the high-PUEM condition (*M* = 5.51, *SD* = 1.12) reported significantly higher levels of perceived upward economic mobility compared to those in the PUEM-control condition (*M* = 4.59, *SD* = 1.49), *t*(777) = 7.52, *p* < .001, Cohen’s *d* = 0.66 with a 95% CI = [0.45, 0.87]. Similarly, their perceptions of upward economic mobility were significantly higher than those in the low-PUEM condition (*M* = 3.59, *SD* = 1.55), *t*(777) = 15.64, *p* < .001, Cohen’s *d* = 1.37 with a 95% CI = [1.61, 1.58]. Additionally, participants in the PUEM-control condition perceived significantly higher upward economic mobility than those in the low-PUEM condition, *t*(777) = 8.12, *p* < .001, Cohen’s *d* = 0.71 with a 95% CI = [0.50, 0.92]. These results suggest that the manipulation of perceived upward economic mobility was effective.

##### Test of the mediating effect of perceived upward economic mobility within the PUEM-control condition

A Mediation analysis was performed according to the strategy recommended by Yzerbyt et al. (2018), with self-esteem and demographic variables included as control variables.^2^ Results suggested that within the PUEM-control condition, relative deprivation negatively predicted perceived upward economic mobility, β = –.41 with a 95% CI = [–.52, –.32], SE = .06, *t* = –7.49, *p* < .001. In addition, perceived upward economic mobility positively predicted self-improvement, β = .72 with a 95% CI = [.63, .82], SE = .05, *t* = 14.64, *p* < .001. These findings suggest a substantial mediating effect of perceived upward economic mobility. A percentile bootstrap analysis with 5000 resamples confirmed the significance of the mediating effect, which was quantified at –.30 with a 95% CI = [–.40, –.20]. These results supported Hypothesis 2.

##### The main effects of RD and PUEM, and the interaction pattern of RD × PUEM

After controlling for self-esteem and demographic variables, a 2 (RD: high vs. low) × 3 (PUEM: high vs. low vs. control) analysis of covariance was conducted.

The main effect of RD manipulation on self-improvement was significant, *F*(1, 769) = 20.50, *p* < .001, partial η^2^ = .03 with a 90% CI = [.01, .05]. As expected, participants in the low-RD condition (*M* = 5.15, *SD* = 1.21) demonstrated a significantly greater tendency towards self-improvement compared to those in the high-RD condition (*M* = 4.57, *SD* = 1.62), *t*(773) = 4.07, *p* < .001, Cohen’s *d* = 0.30 with a 95% CI = [0.15, 0.44].

The main effect of PUEM manipulation on self-improvement was also significant, *F*(1, 769) = 57.23, *p* < .001, partial η^2^ = .13 with a 90% CI = [.09, .17]. Participants in the high-PUEM condition (*M* = 5.46, *SD* = 1.10) reported significantly greater levels of self-improvement compared to those in the PUEM-control condition (*M* = 4.87, *SD* = 1.42, *t*(772) = 5.06, *p* < .001, Cohen’s *d* = 0.45, 95% CI = [0.23, 0.66]. Similarly, their self-improvement scores were significantly higher than those in the low-PUEM condition (*M* = 4.25, *SD* = 1.55), *t*(772) = 10.48, *p* < .001, Cohen’s *d* = 0.93, 95% CI = [0.72, 1.14]. The difference between the PUEM-control and low-PUEM condition was also significant, *t*(772) = 5.46, *p* < .001, Cohen’s *d* = 0.48 with a 95% CI = [0.27, 0.70].

**Figure 3:**
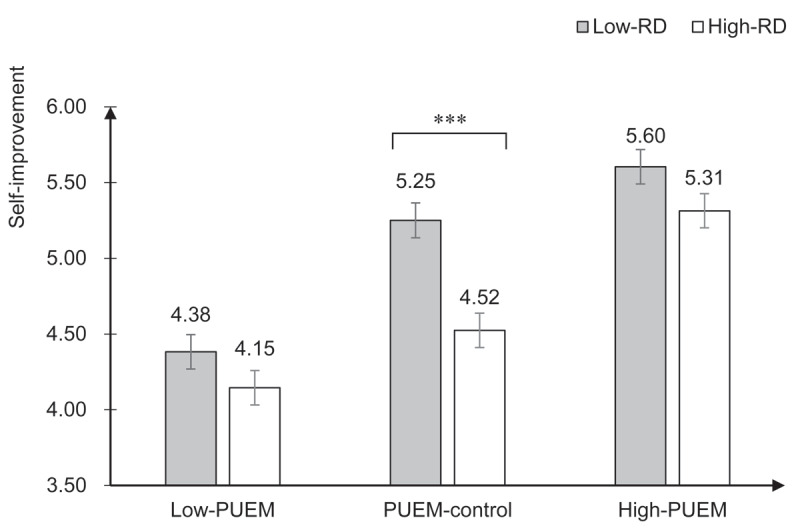
Means of self-improvement in different conditions (Study 3). *Notes*. RD: relative deprivation, PUEM: perceived upward economic mobility. Means are adjusted for covariates. Error bars represent ±1 *SE* of the means. ***p < .001.

More importantly, the results revealed a marginally significant interaction effect between RD and PUEM manipulation on self-improvement, *F*(1, 769) = 2.92, *p* = .055, partial η^2^ = .01 with a 90% CI = [.000, .02]. As illustrated in Figure 3, a simple effect analysis indicated that under the PUEM-control condition, relative deprivation significantly and negatively impacted self-improvement, *F*(1, 769) = 21.00, *p* < .001, partial η^2^ = .03 with a 90% CI = [.01, .05]. In contrast, under the mediator-blocking conditions, the effect of relative deprivation on self-improvement diminished markedly and became non-significant. Specifically, in the low-PUEM condition, *F*(1, 769) = 2.24, *p* = .14, partial η^2^ = .003, 90% CI = [.000, .013], and in the high-PUEM condition, *F*(1, 769) = 3.44, *p* = .06, partial η^2^ = .004, 90% CI = [.000, .016], the impact of relative deprivation was substantially reduced.

To further explore the interaction, we conducted additional contrast analyses with the high-RD and low-RD conditions separately.^3^ For the high-RD condition, we first assigned contrast codes (–2, 1, 1) to the high-PUEM, PUEM-control, and low-PUEM conditions, respectively, to examine whether participants in the high-PUEM condition reported significantly higher levels of self-improvement compared to the other two conditions combined. Results indicated a significant difference, suggesting that despite high relative deprivation, participants in the high-PUEM condition showed greater self-improvement. The results showed that despite the high level of relative deprivation, participants in the high-PUEM condition still showed greater self-improvement compared to those in the other conditions, estimate = 1.96, SE = 0.27, 95% CI = [1.27, 2.64], *t*(769) = 7.18, *p* < .001. Next, we assigned contrast codes (0, –1, 1) to directly compare the low-PUEM condition with the PUEM-control condition. Participants in the low-PUEM condition reported marginally lower self-improvement than those in the PUEM-control condition, estimate = –0.38, SE = 0.16, 95% CI = [–0.77, 0.02], *t*(769) = –2.41, *p* = .07.

For the low-RD conditions, contrast codes (–1, –1, 2) were used to compare self-improvement across the high-PUEM, PUEM-control, and low-PUEM conditions. The results indicated that although relative deprivation was low, participants in the low-PUEM condition reported significantly lower levels of self-improvement compared to the other conditions, estimate = –2.09, SE = 0.27, 95% CI = [–2.77, –1.41], *t*(769) = –7.69, *p* < .001. Additionally, contrast codes (–1, 1, 0) compared the high-PUEM condition directly with the PUEM-control condition. Participants in the PUEM-control condition demonstrated slightly lower levels of self-improvement compared to the high-PUEM condition, although this difference was not statistically significant, estimate = –0.36, SE = 0.16, 95% CI = [–0.75, 0.03], *t*(769) = –2.26, *p* = .10.

These results indicated that the effects of relative deprivation on self-improvement were reduced or even eliminated when perceived upward economic mobility was blocked experimentally (i.e., held constant through manipulation), providing support for Hypothesis 2. Specifically, this finding suggests that perceived upward economic mobility mediates the relationship between relative deprivation and self-improvement. Furthermore, participants in the high-PUEM condition demonstrated significantly greater self-improvement than those in both the PUEM-control and low-PUEM conditions, even under conditions of high relative deprivation. Additionally, even when relative deprivation was low, participants experiencing low PUEM reported lower levels of self-improvement compared to individuals in the high-PUEM and PUEM-control conditions. Collectively, these findings highlight the crucial role of perceived upward economic mobility, suggesting that fostering individuals’ hope regarding upward mobility could buffer against the adverse effects of relative deprivation and thereby facilitate sustained self-improvement.

## General Discussion

Relative deprivation is a pervasive phenomenon (Haisley, Mostafa & Loewenstein 2008; Osborne & Sibley 2013; Zoogah 2010), making it essential to explore how individuals respond to such feelings. The current study investigated the relationship between relative deprivation and self-improvement behaviors, proposing that perceived upward economic mobility acts as the underlying mechanism through which relative deprivation inhibits engagement in self-improvement activities. Utilizing both survey and experimental data, this research provides robust evidence that relative deprivation negatively affects individuals’ perceptions of upward economic mobility, thereby hindering their intentions to pursue self-improvement. Notably, as demonstrated in Study 3, interventions targeting perceived upward economic mobility can mitigate the adverse effects of relative deprivation.

Our results align with existing research highlighting the negative effects of economic inequality. For instance, McCall et al. (2017) demonstrated that participants believed that the economic inequality in their society was high and increasingly expressed greater skepticism about their ability to achieve upward mobility. Additionally, Bak and Yi (2020) found that perceived inequality diminishes future-oriented behaviors by affecting individuals’ perceptions of economic mobility.

However, Zoogah (2010) argued for an overall positive effect of relative deprivation on participation in development activities (i.e., intention to attend university MBA courses), a form of self-improvement. The inconsistency between the current study’s findings and those of Zoogah may stem from differences in measurement. In Zoogah’s research, participants reported their cognition and resentment of payment gaps between foreign-educated employees and themselves, exemplified by items like ‘Foreign-educated employees are paid more.’ This framing may have led participants to attribute their disadvantages to their educational background, suggesting a clear path for improvement through further education. In Ghana, where Zoogah conducted the research, opportunities for university enrollment are limited, making MBA courses a valued educational pursuit and a symbol of upward mobility. Consequently, feelings of relative deprivation prompted greater intentions to engage in such kinds of development activities. The specificity of the comparison to foreign-educated employees likely encouraged participants to attribute their situation to personal deficiencies while simultaneously identifying a means to achieve upward economic mobility.

The results of the current study suggest that relative deprivation can create a vicious cycle, offering valuable insights into why individuals in disadvantageous situations often deteriorate further. Specifically, those who feel relatively deprived may perceive their chances of changing their circumstances as low, leading to decreased motivation for self-improvement, which can exacerbate their situation. Fortunately, the findings from Study 3 reveal a potential intervention: enhancing individuals’ perceptions of upward economic mobility can help disrupt this cycle. When people believe they have a high level of upward economic mobility, they are more willing to engage in self-improvement activities, regardless of their feelings of relative deprivation.

However, it is crucial to caution against the potential pernicious effects of unrealistic perceptions of upward economic mobility. Overestimating upward economic mobility may foster a prevalence of belief in meritocracy (Day & Fiske 2017), which can lead to undue blame on the economically disadvantaged for their hardships (Hoyt et al. 2021). At the societal level, this overestimation may cause individuals to defend problematic systems (Day & Fiske 2017), increase tolerance of economic inequality (Shariff, Wiwad & Aknin 2016), and reduce support for policies aimed at reducing economic disparities (McCall et al. 2017). Therefore, policymakers should develop strategies that genuinely improve upward economic mobility within the societal system, enabling people to perceive an actual increase in upward mobility.

While a heightened perception of upward economic mobility can mitigate the negative impact of relative deprivation on self-improvement, it is essential not to overlook the issue of relative deprivation itself. Prolonged feelings of relative deprivation can adversely affect self-regard, well-being, and life satisfaction (e.g., Osborne & Sibley 2013). Therefore, it is crucial to address relative deprivation directly through targeted interventions alongside efforts to enhance perceptions of upward mobility.

Despite its theoretical and practical implications, the current research has several limitations. Firstly, our focus was primarily on economic relative deprivation, which is particularly salient in today’s context of rising economic inequality (Liu, Sun & Bao 2024; Osborne, García-Sánchez & Sibley 2019). However, other forms of relative deprivation exist, such as those related to social relationships (Bolino & Turnley 2009) and careers (Buunk et al. 2003). Future studies should not only assess the applicability of our findings to these other domains but also explore potential spillover effects. For instance, does relative deprivation in one area impede self-improvement behaviors in another?

Secondly, we speculate that relative deprivation may create a vicious cycle. Individuals who experience relative deprivation often believe their unfavorable situations are difficult to change, leading to a lack of motivation for self-improvement. This demotivation can ultimately result in a deeper disadvantage. However, the current research does not fully explore this cycle. Future studies should adopt a social-ecological perspective to address this gap and empirically investigate the consequences of choosing not to engage in self-improvement behaviors when faced with adverse circumstances.

Thirdly, the current research focused on self-improvement intention rather than actual self-improvement behaviors. While intention is closely related to behaviors, they are not synonymous, as highlighted by the Theory of Planned Behavior (Ajzen 1991). We recommend that future research conduct time-lagged studies to examine whether increases in feelings of relative deprivation correspond with a decline in actual self-improvement behaviors. Additionally, the manipulation of relative deprivation was based on scenarios and imagination, which may limit the ecological validity and practical significance of the findings. Future studies should employ more ecologically valid methods to manipulate relative deprivation and assess its effects.

Fourthly, the current research examined the effects of relative deprivation without considering potential individual differences. Not everyone responds to a state of relative deprivation with hopelessness; some individuals actively strive to improve their circumstances (Zoogah 2010). We recommend that future studies investigate the moderating role of individual differences, such as a growth mindset—the belief that talents can be developed through effort, effective strategies, and guidance (Yeager & Dweck 2020). In contrast, those with a fixed mindset view their talents as inherent traits. For example, a study of high school students found that those from low-income families who possessed a growth mindset achieved academic performance comparable to that of their higher-income peers (Claro, Paunesku & Dweck 2016). These findings suggest that in disadvantaged situations, a growth mindset may serve as crucial psychological capital, motivating individuals to take action for self-improvement. Conversely, individuals with a fixed mindset may lack such internal resources and be more prone to give up.

## Conclusion

In conclusion, our findings contribute to existing theories and research on the impact of relative deprivation on self-improvement behaviors. The experience of relative deprivation prompts individuals to question their prospects for upward mobility, which subsequently hinders their engagement in self-improvement behaviors. This mediating mechanism has been consistently demonstrated across four studies highlighting the importance of addressing perceptions of upward economic mobility in efforts to foster self-improvement.

## Data Accessibility Statement

The dataset and syntax, as well as the Supplementary Materials can be found by accessing the OSF link below (Files tab): https://osf.io/rdkx3/.

## Footnotes

Although we had preregistered that self-worth would be treated as a covariate, it was actually measured using four items adapted from the Rosenberg Self-Esteem Scale. Thus, we concluded it was more accurate to directly refer to this construct as ‘self-esteem’. This revision was consistently applied to Study 3 as well.

Although our preregistration specified testing the effect of relative deprivation on perceived upward economic mobility within the PUEM-control condition, we subsequently determined that testing the mediating role of perceived upward economic mobility would offer more informative insights into our hypothesized relationships.

Although these additional analyses were not included in our preregistration, we believe they provide valuable supplementary information regarding the effects of our experimental manipulations. Therefore, we have chosen to incorporate them into our revised manuscript.
